# The Role of Nodes and Nodal Assessment in Diagnosis, Treatment and Prediction in ER+, Node-Positive Breast Cancer

**DOI:** 10.3390/jpm13101476

**Published:** 2023-10-08

**Authors:** Charlene Kay, Carlos Martinez-Perez, J. Michael Dixon, Arran K. Turnbull

**Affiliations:** 1 Translational Oncology Research Group, MRC Institute of Genetics and Molecular Medicine, Western General Hospital, University of Edinburgh, Edinburgh EH4 2XU, UK; 2 Edinburgh Breast Unit, Western General Hospital, NHS Lothian, Edinburgh Eh4 2XU, UK

**Keywords:** breast cancer, lymph nodes, oestrogen receptor, endocrine therapy, recurrence, node-positive breast cancer, metastasis, biomarkers, predictive tools, prognosis

## Abstract

The majority of breast cancers are oestrogen receptor-positive (ER+). In ER+ cancers, oestrogen acts as a disease driver, so these tumours are likely to be susceptible to endocrine therapy (ET). ET works by blocking the hormone’s synthesis or effect. A significant number of patients diagnosed with breast cancer will have the spread of tumour cells into regional lymph nodes either at the time of diagnosis, or as a recurrence some years later. Patients with node-positive disease have a poorer prognosis and can respond less well to ET. The nodal metastases may be genomically similar or, as is becoming more evident, may differ from the primary tumour. However, nodal metastatic disease is often not assessed, and treatment decisions are almost always based on biomarkers evaluated in the primary tumour. This review will summarise the evidence in the field on ER+, node-positive breast cancer, including diagnosis, treatment, prognosis and predictive tools.

## 1. Background

Breast cancer (BC) makes up 15% of all cancer cases, with an estimated 287,850 new cases of BC diagnosed in the United States in 2022 [1] and 55,900 in the United Kingdom [2]. Despite many therapeutic advances in treating BC, it still accounts for 7% of all cancer deaths, taking the lives of 685,000 individuals globally in 2020 alone [3].

Although BC is one of the most extensively studied types of cancer and treatment options have expanded widely in recent years, many challenges remain. One crucial unmet challenge is the development of drug resistance, which occurs in many oestrogen receptor-positive (ER+) breast cancers and can eventually lead to death in some patients.

More than 70% of all breast cancers are hormone receptor-positive (HR+) at diagnosis [4], meaning they overexpress the oestrogen receptor (ER), progesterone receptor (PR) or both. As these tumours are driven by oestrogen, they are likely to respond to endocrine therapy (ET) that blocks oestrogen signalling. The selective oestrogen receptor modulator (SERM) tamoxifen is frequently used to treat BC in premenopausal women and an aromatase inhibitor (AI), such as letrozole, anastrozole or exemestane, is the most common drug used in postmenopausal women. Goserelin, a gonadotropin-releasing hormone (GnRH) agonist, is also often used in higher risk premenopausal women to suppress ovarian function. The vast majority of HR+ BCs are human epidermal growth factor receptor 2 (HER2)-negative (HER2-), with less than 10% of HR+ tumours being HER2-positive (HER2+) in addition to overexpressing ER and/or PR [5,6]. These tumours can be more complex, being driven by HER2, ER signalling or both, so the focus of this review will be on ER+/HER2-BC.

One high-risk group is patients who, in addition to cancer in the breast, also have metastatic spread in one or more of their regional lymph nodes. Nearly one third of those diagnosed with primary BC will also have tumour in their lymph nodes at the time of diagnosis [1]. These patients have a poorer prognosis than node-negative patients [7] and often require more intensive treatment.

As is the case with many cancer types, BC metastasises through the bloodstream or through lymphatics to regional lymph nodes and from there to other organs [8]. Once BC has spread beyond the regional lymph nodes to other nodal groups, bone, liver, lung, brain or other organs, the cancer is considered metastatic BC and is incurable. Identifying patients with involved axillary lymph nodes and surgically removing them and/or treating with local therapies such as radiotherapy or systemic therapies is key to stopping spread to other organs.

The aim of this review is to provide a comprehensive bank of knowledge on the topic of ER+/HER2- node-positive BC, including diagnosing nodal involvement, its effect on prognosis, evaluating treatment options and using gene signatures to predict treatment response to systemic therapies.

## 2. Diagnosis

**Figure 1:**
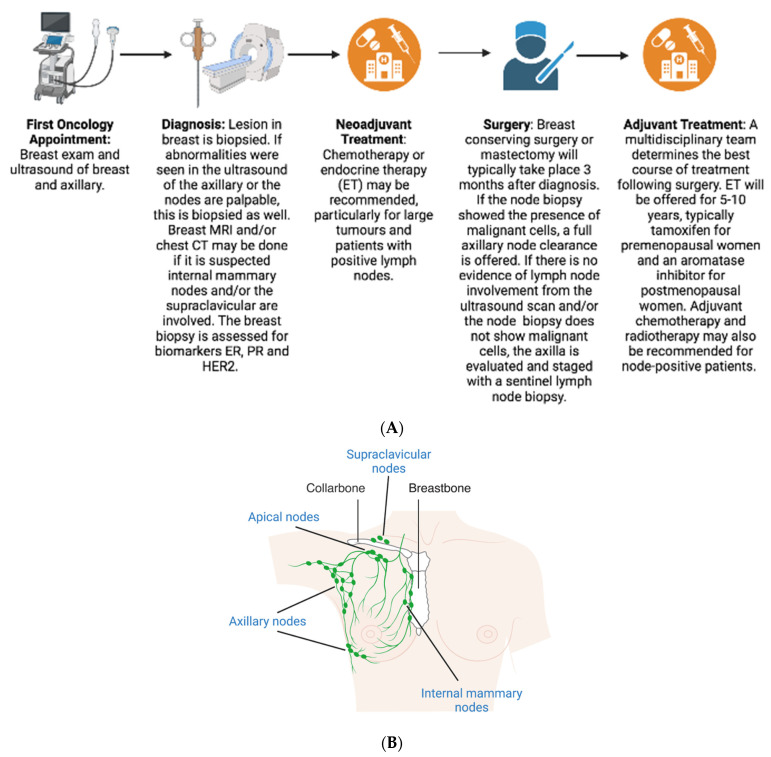
Triple Assessment. (**A**) The breast cancer clinical care pathway, in which physical examination, breast imaging and biopsy take place. (ER = oestrogen receptor, PR = progesterone receptor, HER2 = human epidermal growth factor receptor.) (**B**) The most common sites of first spread of breast cancer. Axillary, apical, supraclavicular and internal mammary nodes in the breast.

When a patient is seen at a breast clinic with suspected BC, they undergo what is referred to as triple assessment—physical examination, breast imaging and biopsy (Figure 1A). A core biopsy of the breast abnormality is taken, and the presence or absence of invasive cancer is determined. The axillary lymph nodes are evaluated by ultrasound, and if any abnormalities are seen, a biopsy of the nodal tissue is also taken. A breast MRI or chest CT scan may also be recommended if it is suspected that internal mammary nodes and/or the supraclavicular are involved (Figure 1B) [9]. If the axillary biopsy shows the presence of malignant cells and pathologically proven lymph node metastasis, then the options include full axillary node clearance, also known as an axillary lymph node dissection (ALND), radiotherapy to the axilla or, if patients have only one or two nodes involved and fulfil Z0011 criteria observation, sentinel lymph node biopsy (SLNB) only [10,11]. If there is no evidence of lymph node involvement from the ultrasound scan and/or the pre-operative biopsy does not reveal malignant cells in the axillary nodes, it is recommended the axilla should be evaluated and staged with SLNB rather than performing an ALND. Nodal metastases are identified by SLNB in up to one third of patients who have a negative axilla on ultrasound [12].

Historically, the vast majority of patients have had all their axillary lymph nodes removed via ALND at the time of breast surgery [13], often removing 10–20 lymph nodes. However, the results of the sentinel node biopsy trials and the American College of Surgeons Oncology Group’s (ACOSOG) Z0011 trial changed this. This randomised phase 3 clinical trial included 856 women with T1 or T2 invasive cancer, of whom 446 had a SLNB alone and 446 had a SLNB followed by an ALND. The results showed no significant difference in 10-year overall survival between the two groups of patients with no palpable lymph nodes and 1 or 2 metastatic lymph nodes on SLNB, thus discouraging routine use of ALND [14]. ALND can cause side effects and complications, including lymphedema, numbness and infection so for many this was a welcome change.

The sentinel lymph node(s) are defined as the first lymph nodes cancer cells are most likely to spread to from the primary lesion [8]. The identification and removal of several sentinel nodes is routine but is surgeon-dependent [15]. In the majority of cases there is more than a single sentinel node. There are a variety of methods that can be used to identify sentinel nodes. These include the use of isotope and blue dye. Intradermal injection of isotope appears to produce the greatest uptake of radioactivity in the nodes. Blue dye has the complication of anaphylaxis manifested by oedema, erythema, tachycardia, “blue” hives, bronchospasms, dysrhythmias, vasodilation, and less commonly cardiovascular collapse. Skin staining with blue dye is another issue for some patients. Blue dye in addition to isotope is now often limited to patients who have had neoadjuvant chemotherapy. Indocyanine green and magtrace are other agents that can be used for SLNB. Both require special detection systems. Issues with magtrace includes cost, brown skin staining and problems using MRI for a period after injection. To circumvent these problems, the magtrace is injected deep in the breast and around the tumour. Much of the magtrace is then removed at surgery. Previous studies have shown that peritumoral injection of tracer is not the optimal site of tracer injection [16]. Magtrace does allow SLNB to be performed at a later date, such as if a patient were having a mastectomy for ductal carcinoma in situ (DCIS) but invasive disease was also found on pathology, necessitating a SLNB. Following tracer injection, the surgeon detects the nodes containing tracer using vison or a detection system. Once the surgeon identifies the sentinel node (or nodes), these are removed. This is most often performed at the time of breast-conserving surgery (lumpectomy or wide local excision) or mastectomy. The optimal number of sentinel nodes is unclear, but one study suggested that better survival outcomes are seen with patients having 3 sentinel nodes removed [17].

The use of SLNB in the over-70 population has been debated, and there is acceptance that SLNB may not be necessary if the patient has clinically and ultrasound-negative axilla and HR+ early BC [18,19]. Patients of this age often have comorbidities and are at higher risk of adverse events following SLNB, and findings from a SLNB are unlikely to change the recommended adjuvant therapy [20]. SLNB and ALND have a risk of complications. The most common is numbness under the arm initially following surgery. This, and lymphedema, may persist for quite some time following surgery and can be very uncomfortable for the patient [21].

If there is no cancer present on pathological assessment, then the patient has lymph node-negative disease and no further axillary treatment is recommended [22]. If only isolated tumour cells (ITC) or micrometastases are present, the patient is also considered to have node-negative BC, and no further axillary treatment is suggested [10]. Disease in lymph nodes smaller than or equal to 0.2 mm are considered ITC deposits and do not influence prognosis [23]. A micrometastasis is defined as a metastatic lesion between 0.2 mm and 2 mm in diameter, whilst a macrometastasis is a metastatic lesion larger than 2 mm in diameter [24,25,26,27]. New methods are being trialled to accurately assess both micro and macrometastases in BC, including the use of indocyanine green [28] and near infrared imaging of tumour-associated macrophages in vivo [29].

Evidence that micrometastases have less impact prognostically is supported by a recent multi-centre study of sentinel node micrometastases in ER+ early BC [30], although other studies have suggested otherwise [27]. ITCs and micrometastases do not have the power to predict recurrence for the individual patient; however, when grouped into large analysis cohorts, metastatic tumour burden has been shown to be a continuous prognostic variable and thus their significance should not be overlooked [27]. A recent study carried out at the University of Wisconsin School of Medicine and Public Health supports this, as patients in their cohort who had grade 3 cancers with micrometastases were shown to be at significant increased risk of locoregional recurrence, and it was suggested they should be offered nodal radiotherapy as a result [31].

If the SLNB reveals one or more macrometastases, further axillary treatment such as clearance or radiotherapy may be offered [10]. There are differing opinions and options about what is appropriate management in women with 1 or 2 lymph nodes that contain macrometastases. In the United Kingdom, the National Institute for Health and Care Excellence (NICE) recommends holding a discussion with the patient to outline the risks and benefits of additional axillary treatment in addition to breast-conserving surgery, whole-breast radiotherapy and systemic therapy. In the United States, the American Cancer Society recommends women with breast tumours 5 cm or smaller and no more than 2 positive sentinel nodes who are having breast-conserving surgery followed by radiation can safely avoid an ALND, as can women with lymph nodes that have no more than 2.0 mm cancer who are undergoing a mastectomy [32].

Once the number of positive lymph nodes is identified, the Tumour, Node, Metastasis (TNM) classification can be determined. In TNM staging, the ‘T’ refers to the size of the primary tumour, ‘N’ gives an indication of how many positive lymph nodes the patient has, and ‘M’ indicates the presence or absence of distant metastasis (Table 1). This anatomic staging classification has been used since the late 1970s, and the most recent iteration even incorporates biomarker status of ER, PR, HER2 and grade as measured in the diagnostic biopsy. The TNM staging plus these four biomarkers now make up the Clinical Prognostic Stage Group, an evaluation determined before any treatment is given [33]. Once patients undergo surgery to remove the primary tumour, a Pathological Prognostic Stage can be determined using ‘T’ and ‘N’ status from surgery. If neoadjuvant treatment was given, post-neoadjuvant status can be indicated as ‘ypT’ and ‘ypN’, where ‘y’ indicates it is post-neoadjuvant therapy and ‘p’ indicates it is a pathological assessment. The 21-gene assay Oncotype DX is also included in current TNM guidelines although only for node-negative patients. Isolated tumour cells found by immunohistochemistry (IHC), molecular findings using RT-PCR and micrometastases can also be noted within TNM staging, with pN0 (i+), pN0 (mol+) and pN1mi, respectively [26]. The American Joint Committee of Cancer (AJCC) further defines the isolated tumour classification pN0 (i+) as ITCs totalling less than 200 and pN1mi as a metastasis greater than 0.2 mm but less than 2.0 mm and/or more than 200 isolated tumour cells.

It has long been clear that an increased number of positive nodes is associated with a worse prognosis [34,35,36,37]. More recently, the importance of lymph node ratio (LNR) has also been investigated. LNR is defined as the ratio of the number of positive nodes to the number of total nodes excised, with a higher ratio being associated with a shorter disease-free survival and overall survival [38]. The rate of locoregional recurrence and distant recurrence are also increased with a higher LNR [39]. LNR may be a better predictor of prognosis than traditional ypN status, but of course this relies on the patient having undergone an ALND and not just SLNB [40,41].

## 3. Pathological Assessment

While a number of different biomarkers have proven useful in prognostic and predictive gene signatures, the receptors ER, PR and HER2 remain key clinical biological markers despite it being several decades since their first adoption [42]. Ki67, a measure of proliferation, is also often assessed. Treatment decisions are made based on the levels of these biomarkers in the primary lesion at diagnosis and they are not typically re-evaluated at the time of surgery when the tumour is removed, or in the nodal tissue if positive nodes are also removed. It should also be noted that biopsies are a snapshot of the tumour only, and whilst they can reveal the presence or absence of cancer, it should not be assumed the entire tumour is biologically identical. BC can be incredibly heterogenous—not just between different patients but within a single tumour, making treatment decisions difficult at times [43,44].

The presence or absence of the hormone receptors ER and PR is assessed by IHC in the diagnostic biopsy and assigned an Allred score of 0–8 by a pathologist. A score of 0 indicates no staining, and a score of 8 describes strongly positive staining in two thirds to 100% of cells throughout the section. A score for HER2 is also determined, first by IHC (0–3+) and then by fluorescence in situ hybridization (FISH) if the IHC score was intermediate (2+). If the FISH score is above a threshold of 2.2, the HER2 gene is considered amplified, and the cancer deemed HER2+. Assessment of the primary tumour’s receptor status as described above is a well-established standard of practice, and scores for these biomarkers are instrumental in guiding treatment selection. However, it is not common practice to also stain cancerous nodal tissue in node-positive patients alongside the primary lesion despite possible discrepancy between primary breast cancers and their nodal metastases [45].

## 4. Treatment

### 4.1. Neoadjuvant

Treatment decisions for BC are made by a multidisciplinary team, especially for higher risk, node-positive patients. The patient’s age, health, comorbidities, and BC-specific factors, including how many positive nodes are present, help determine these treatment decisions. Treatment guidelines vary depending on the BC subtype (HR+/HER2-, HR+/HER2+, HR-/HER2+, or triple-negative) and patients with positive nodes are more likely to be candidates for neoadjuvant and adjuvant chemotherapy. For many patients with node-positive disease, neoadjuvant treatment is used to downstage the tumour and reduce the amount of surgery required.

Neoadjuvant chemotherapy (NAC) can downsize and downstage both the primary tumour and the axilla, and greatly reduce the need for ALND in clinically N1 patients [46]. Neoadjuvant endocrine therapy (NET) is also an option for node-positive patients [47], although less utilised globally than NAC. A recent review of studies that included ER+/HER2- node-positive patients treated with NET revealed an axillary pathological complete response (pCR) rate of 14.48%, notably higher than the 10% previously reported [48].

### 4.2. Surgery

In the UK, NICE guidelines recommend performing SLNB rather than axillary clearance if there is no evidence of axillary involvement via ultrasound or the axillary biopsy was negative [10]. There has been some debate on the number of nodes that need to be removed in a SLNB to definitively say the axilla is negative. However, a study of the SEER database revealed improved survival outcomes in patients with three or more nodes removed, in particular contrast to the poorer outcome seen in patients with only one sentinel node removed [11,17].

The Z0011 trial was instrumental in bringing about change in axillary clearance rates, as it showed no significant difference in survival between patients with no palpable lymph nodes and 1 or 2 metastatic lymph nodes after sentinel node assessment, granting these patients a reprieve from further surgery [14]. Those with micrometastases or isolated tumour cells in their sentinel nodes are treated as node-negative and also do not gain benefit from further axillary treatment, as supported by data from the AATRM trial [10,49].

Full axillary clearance, or axillary radiotherapy if the patient is to undergo a mastectomy, are suitable options for patients with positive lymph nodes following SLNB. Despite equivalent disease-free survival and overall survival, axillary radiotherapy is underused compared to ALND yet remains a suitable option for some [11,50]. Overall, doing less in the axilla is the sensible route forward as we continue to improve predictive methods.

### 4.3. Adjuvant

Following surgery, adjuvant therapy options are discussed by a multidisciplinary team and the NHS Predict tool can be used to estimate prognosis and predict benefit from adjuvant therapies. Typically, men and premenopausal women with ER+ BC are first offered adjuvant tamoxifen, while postmenopausal women receive an AI. Treatment for ER+ breast cancers is now frequently offered for longer than the standard 5 years of adjuvant treatment, in particular for those who are deemed higher risk for recurrence, such as those with node-positive disease [51]. This may be either extended (longer than 5 years) tamoxifen therapy or switching to an AI after 5 years of tamoxifen.

While the majority of patients with ER+ BC typically respond well to ET initially, resistance to endocrine therapies is a significant problem. Approximately 10% of ER+ cancers will be intrinsically resistant, and in 30–40% of patients, their tumours acquire resistance over time [52,53]. When these patients acquire resistance and the cancer recurs, it may recur locally in the breast, regionally in the axillary lymph nodes, or in other distant sites, emphasising the need for a better understanding of endocrine resistance [54].

The patient may also be offered adjuvant chemotherapy if the risk of recurrence is deemed high enough based on the tumour profile, the extent of any axillary nodal involvement, risk assessment using Predict, or tumour profiling using tests like Oncotype DX. Some node-positive patients may be able to avoid chemotherapy in the adjuvant setting. A recent study in South Korea reported similar survival statistics between patients who received only ET and patients who received both ET and chemotherapy [55]. Notably, these were all patients with stage N1 disease, and therefore had a maximum of three positive lymph nodes. A recent study utilising the Oncotype DX test, previously used only for node-negative patients, also showed some node-positive patients are likely being overtreated with adjuvant chemotherapy, and for some, it is not necessary [56]. The side effects associated with chemotherapy are typically much worse than those associated with ET; therefore, identifying patients who do and do not require chemotherapy is essential.

Adjuvant bisphosphonate therapy is also recommended for postmenopausal women with node-positive invasive BC to help prevent or delay bone health decline, bone pain, and recurrence in the bone [57]. Most node-positive BC patients will be offered adjuvant radiotherapy after breast-conserving surgery, and chest wall radiotherapy is delivered to women at high risk of local recurrence. If the patient has one to three positive lymph nodes (N1) and other poor prognostic factors such as high histological grade, or there are four or more positive axillary lymph nodes (N2–3), adjuvant radiotherapy to the supraclavicular fossa is offered according to NICE guidelines NG101 [10]. If an axillary clearance has already been carried out, adjuvant radiotherapy to the axilla is not recommended unless extensive nodal burden remains following clearance. Finally, the internal mammary nodal chain should also be considered for adjuvant radiotherapy treatment in node-positive patients.

## 5. Prognosis

The 5-year survival rate for localised invasive BC, defined as disease in the breast only, is over 99%; however, only 63.5% of patients are confirmed as having disease solely in the breast at the time of diagnosis. Overall, 29% of patients have cancer present in their regional lymph nodes when diagnosed, and 6% have distant metastases. The 5-year survival rate drops to 86.1% and 30.0% for regional and distant disease, respectively [1].

Node-positive BC comes with a 31% risk of distant metastasis within 20 years of primary diagnosis if initially diagnosed with 1–3 positive nodes and jumps to 52% in those with 4–9 positive nodes. The 20-year risk of death is 15%, 28% and 49% for patients with node-negative (N0), 1–3 (N1) and 4–9 positive nodes (N2), respectively [37]. These significant differences in survival highlight the crucial importance of being able to accurately predict response and recurrence in those with node-positive BC.

## 6. Prediction

Predicting treatment response in patients with node-positive BC aids in decision making conversations with their oncologist, facilitates more personalised care, and ultimately improves survival outcomes. There are a number of predictive tools used in BC care, some more accurate than others in node-positive disease (Table 2). Because patients with node-positive BC at diagnosis make up a minority of cases and have been studied less, predicting who within this group will respond well to ET and other adjuvant treatments has been difficult, resulting in both over and under-treatment of different subgroups [58].

In addition to the one third of patients with BC with node-positive status at diagnosis [1,59], a number of patients go on to develop nodal recurrences whilst on ET or after ET has finished. There is a clear unmet need for reliable and accurate predicative models to predict response and recurrence in ER+ node-positive BC both at the time of diagnosis and whilst on treatment.

### 6.1. Predict

The online Predict tool [60], endorsed by the AJCC, is often used in treatment decision making in early invasive BC to predict the survival outcome if one or several treatments are given [61]. It was originally developed using data from 5000 women with BC and later validated with data from 23,000 women. The tool is used routinely in the UK and has now been validated in BC patients in the United States [62]. In 2016, this web tool was accessed over 20,000 times per month from all around the world with likely increased numbers today [63]. It is now on version 2.2. The tool considers tumour size, ER, HER2 and Ki67 status, the number of positive nodes, the patient’s age and menopausal status. Predict is often used by an oncologist together with their patient so that post-surgery treatment options and their associated benefit can be discussed. The estimated benefit from 5 or 10 years of ET, adjuvant chemotherapy and bisphosphonates (if the patient is postmenopausal) is generated, and that information can then be used to make treatment decisions. Predict has recently been independently validated in a Scottish cohort of more than 45,000 patients and deemed still relevant for patients today [64], although there are some limitations. It is not designed to be used for patients with bilateral disease or DCIS and it does not account for newer treatments such as CDK4/6 inhibitors, nor does it predict benefit from radiotherapy.

### 6.2. Oncotype DX

Oncotype DX is another prediction tool often used in BC care. This predictive gene test is specifically for use in ER+/HER2- disease to predict adjuvant chemotherapy benefit. Given the well-known side effects from chemotherapy [65,66,67], this toxic treatment should be avoided for some patients when it is safe to do so.

Oncotype DX, also a prognostic indicator, uses real-time reverse-transcription polymerase chain reaction (RT-PCR) to evaluate gene expression levels of 21 genes (16 cancer-related genes and 5 reference) in RNA extracted from formalin-fixed, paraffin-embedded (FFPE) breast tumour tissue. From this data, a recurrence score (RS) between 0 and 100 is generated and predicted chemotherapy benefit and risk of distant recurrence determined. An RS score < 18 is deemed low-risk, RS 18–30 intermediate-risk and RS > 31 high-risk [68].

Oncotype DX has been validated in node-negative BC patients in the TAILORx clinical trial [69] and in node-positive patients in the SWOG-8814 [70] and RxPONDER clinical trials [71]. Chemotherapy plus ET versus ET alone were compared, and the results determined chemotherapy was unnecessary in 70% of women with early BC. More specifically, if a postmenopausal patient has ER+/HER2- BC and an RS of 25 or lower and no more than three positive lymph nodes, they can be spared chemotherapy. The test is not as accurate if the patient has greater than three positive nodes [72]. The cost-effectiveness of using Oncotype DX with node-positive early BC patients has recently been evaluated in both the UK and Canada, and the results showed it is highly likely to be cost-effective, providing further evidence of its use in the node-positive subgroup [73,74].

### 6.3. MammaPrint

The MammaPrint test uses a 70-gene signature related to early disease invasion and metastasis to predict disease outcome and, more specifically, those patients most likely to develop a BC recurrence or metastasis and who therefore require chemotherapy [75,76]. The test is carried out on DNA microarrays. MammaPrint was first evaluated in the RASTER (microarRAy-prognoSTics-in-breast-cancER) study [77], then validated by the prospective randomised MINDACT trial (Microarray In Node-negative Disease may Avoid ChemoTherapy) [78] and, similarly to Oncotype DX, is established as appropriate for node-positive patients with a maximum of three positive nodes. Based on expression levels of its 70-gene signature, MammaPrint stratifies patients into either a genomic low-risk or genomic high-risk group. Women who were considered clinically high-risk by traditional clinical parameters but had a low genomic risk based on gene signature results had a 5-year distant metastasis-free survival (DMFS) of 94.7% after treatment with only ET, indicating no need for chemotherapy in this group. The high-risk group showed significant improvement in BC-specific survival (BCSS) and distant disease-free survival when treated with ET and chemotherapy rather than solely ET, suggesting dual treatment is beneficial in this group [79]. NICE does not currently recommend the use of MammaPrint in the UK as it is not cost-effective (NICE diagnostic guidance DG34) [80].

### 6.4. PAM50 (Prosigna)

The Prediction Analysis of Microarray 50 (PAM50) test, an FFPE RNA-based quantitative RT-PCR assay, was designed to distinguish prognostic significance in known biological subtypes of BC and determine those who would benefit from NAC [81]. In fact, the test can determine which breast cancers are more likely to metastasise as well as identify a low-risk subset of patients unlikely to need chemotherapy [82]. In the UK, the test is now run through NanoString Technologies’ nCounter Digital Analyser.

The PAM50 test identifies a tumour’s biological subtype (Luminal A, Luminal B, HER2-enriched or Basal-like) and generates a risk of recurrence (ROR) score between 0 and 100, which stratifies patients into low-, intermediate- and high-risk groups accordingly. PAM50 has been widely validated prognostically [83,84,85,86,87]. It provides an indication of the patient’s 10-year risk of distant recurrence and can be used with postmenopausal patients who have 0–3 positive lymph nodes. Chemotherapy is recommended in the high-risk group, and not for those in the low-risk group. The recommendations are less clear in the intermediate group, but this stratification can still help guide decision making between the patient and their oncologist. Node-negative patients are considered high-risk if their score is between 61 and 100 and node-positive patients (with up to three positive nodes) are considered high-risk if they have a ROR score between 41 and 100. It has been suggested the test may be improved by the addition of a 13-gene hypoxia signature [88].

Utility of the Prosigna test is currently being tested further in the large-scale OPTIMA (Optimal Personalised Treatment of early breast cancer using Multi-parameter Analysis) trial [89,90]. This multi-site randomised trial will include 4500 patients, some of whom will have 4–9 positive lymph nodes, a cohort less studied previously.

### 6.5. EndoPredict

EndoPredict (EP) is a multigene test that has been proven prognostically successful in establishing both early and late metastatic risk in postmenopausal patients with ER+/HER2- BC [91,92]. The test, which includes a proliferative and oestrogen signalling gene signature with a total of 12 genes, is completed by reverse transcription-quantitative polymerase chain reaction (RT-qPCR). It has also recently been validated in premenopausal women, including those with up to three positive nodes [93]. EP can predict a patient’s risk of distant recurrence in the 10 years following surgery, perhaps more notably the risk of late recurrence in years (up to 15 following surgery) and whether a patient will benefit from chemotherapy or not. EPclin (which combines EP plus two clinical variables—tumour size and nodal status) also generates an individual risk score for each patient.

A low-risk score (EP < 5 or EPclin < 3.3) means that the patient’s cancer is unlikely to recur, and thus, they can safely avoid chemotherapy. A high-risk score (EP ≥ 5 or EPclin ≥ 3.3) indicates a higher likelihood of recurrence, and therefore, chemotherapy is recommended. Notably, node-positive BC patients have a higher risk of late recurrence compared to patients with node-negative disease, hence the need for a late predictor test [92]. EP has also shown concordance with PAM50 in ER+/HER2- node-positive breast cancer, with clinical parameters aiding prognostic proficiency [94] and has aided therapy recommendations in a substantial number of patients [95].

### 6.6. Breast Cancer Index

The Breast Cancer Index (BCI) is a predictive test that can predict recurrence as well as indicate benefit from extended adjuvant ET [96,97,98,99]. The test incorporates 11 genes and can predict a patient’s risk of distant recurrence up to 10 years after surgery. As with most of the predictive gene tests, BCI is validated for node-negative BC patients and node-positive patients with a maximum of three positive nodes.

### 6.7. IHC4

The IHC4 test uses the results of four IHC markers—ER, PR, HER2 and Ki67—to predict 10-year distant recurrence free survival and benefit from chemotherapy in women who have had 5 years of adjuvant ET [100,101,102,103,104]. Clinical and pathological features including the patient’s age, tumour size, tumour grade, nodal status, and type of ET administered for 5 years (tamoxifen vs. AI) are also included in a revised version of the test (IHC4+C) [104]. If adequately sampled, a core biopsy can be used for the test if a whole FFPE section is not available [105]. A modified IHC4 test has also recently been proven prognostically useful for those with metastatic ER+/HER2- BC [106].

## 7. Discussion

Node-positive BC patients are inherently higher risk patients, known to have a worse prognosis compared to patients with node-negative disease. Efforts have been made to better stratify node-positive patients into clearer prognostic and predictive groups to avoid overtreatment. Nevertheless, further improvements in pathological assessment and predictive tools are needed.

A patient with positive lymph nodes is characterised by the number of metastatic nodes they have, and treatment decisions are made based on this number. However, the nodal tissue itself is rarely evaluated for known biological biomarkers in the same way the primary tumour is. This represents a clear missed opportunity. Indeed, the assumption that the cancer in the nodes is biologically identical to the primary tumour is a key caveat and a real limitation of how disease with regional involvement is currently assessed.

It is now well known that there can be discordance between the primary tumour in the breast and the cancer in the nodes [107]. Hormone receptor status may change from HR+ to HR-; the primary cancer may be HER2- whilst the nodal cancer is HER2+; the nodal disease triple-negative whilst its primary tumour was HR+ and so on. There are a number of biological changes that can occur as the cancer progresses to the nodes, though the rate at which this happens is unclear. Some studies have suggested discordance between the primary and nodal cancer happens often—more than 30% for ER, 40% for PR and 24% for HER2 [108]—and others say it is not often enough to warrant evaluating biomarkers in all positive nodes [109,110], and yet others suggest it is somewhere in between [111,112]. To definitively characterise nodal metastases, it may be necessary to evaluate the same biomarkers in cancerous nodal tissue in addition to the primary tumour, as this information could change the treatment plan for some patients. It could also provide a more accurate assessment of future risk of recurrence and, if incorporated into a predictive test, it could identify those who will respond well to a particular treatment and those who may not.

BC is well established as a heterogenous disease, and if it is heterogenous within the primary site, it is likely to be heterogeneous in metastatic sites as well. Heterogeneity together with either natural or treatment-induced biological changes over time can result in biologically very different tumours in the nodes or distant sites compared to the primary lesion [113]. Results from the BOLERO-2 trial indicated just this, as a greater percentage of metastatic tumours were determined to be HER2-enriched compared to their primary cancers, and this influenced prognosis [114]. The assumption that the tumour is still the same or similar enough to the primary tumour could have serious implications if that is not the case. Reasons why every extracted tissue sample is not repeatedly tested for key biomarkers include cost, feasibility and a lack of understanding of the intrinsic and evolved changes to the tumour over time. The identification of additional prognostic and predictive biomarkers specific to lymph node metastases could aid treatment decision making in this cohort of patients.

Predictive gene tests have become useful tools for clinicians when determining the best course of treatment(s) for a patient, specifically in deciding on the administration of chemotherapy or length of adjuvant endocrine therapy. Oncotype Dx, MammaPrint, Prosigna, EndoPredict, the Breast Cancer Index and IHC4 are all recommended by current American Society of Clinical Oncology (ASCO) guidelines [115]. However, they are not without their limitations. In node-positive patients specifically, most predictive gene tests have been well validated for patients with 1–3 positive lymph nodes only; patients with more than three positive lymph nodes have made up such a small percentage of the cohorts studied to date that it is difficult to specifically test its value in this group. Saghachian et al. aimed to change this in 2013 with their study utilising the MammaPrint test in 173 patients with 4–9 positive lymph nodes. Results of the 126 patients in the Luminal A subgroup (ER+, PR+/− and HER2-), of which 66 were deemed low genomic risk and 60 high genomic risk, showed MammaPrint to be significantly prognostic of both DMFS and BCSS in this subgroup [116]. Of course, the algorithms of these tests utilise node status and the number of positive nodes; however, these tests do not directly assess the nodal tissue itself. There is a key need to do this in patients with both few and many nodes involved, given the known heterogeneity of both primary BC and metastatic lesions [117].

Newer prognostic and prediction tests have also been developed [118,119,120]. Some of these newer approaches assess smaller gene panels, which may be more cost-effective globally. However, not all tests include node-positive patients, and still none include direct measurement of nodal metastases, so the need for better predictors remains. Gene expression differences between primary BC and lymph node metastases are probable; however, large-scale studies have yet to be conducted. A recent study of 14 matched primary and lymph node metastases revealed 673 differentially expressed genes, including 348 upregulated genes and 325 downregulated genes [121].

In conclusion, node-positive breast disease is complex and has a higher risk of recurrence than node-negative cancer. There is an unmet need for more studies in patients with more than three positive nodes (i.e., N2 or N3). While this cohort is already established as higher risk due to their nodal status, better stratification and more accurate prediction could enable a more refined treatment selection, such as some patients being able to avoid chemotherapy and/or overtreatment with other adjuvant therapies. Crucially, this will necessitate that predictive tools perform biological assessment of the actual nodal disease, as no predictive gene test currently considers the nodal tissue. In our view, characterisation, biomarker and mutation studies in cancerous nodal tissue is likely to shed light on the mechanisms of metastasis and reveal treatable targets, enabling a move toward better management and improved outcomes in patients with ER+, node-positive BC.

## Figures and Tables

**Table 1:** The Tumour, Node, Metastasis (TNM) system. (T = Tumour, N = Node, M = Metastasis) [33].

**Tumour**		
TX	Primary tumour can’t be assessed	
T0	No sign of a primary tumour	
Tis	Carcincoma in situ (DCIS or Paget’s disease)	
T1	Tumour ≤ 20 mm. There are 4 subcategories of T1:	
	T1mi	Tumour ≤ 1mm
	T1a	Tumour > 1 mm but not >5 mm
	T1b	Tumour > 5 mm but <10 mm
	T1c	Tumour > 10 mm but not >20 mm
T2	Tumour > 20 mm but not >50 mm	
T3	Tumour > 50 mm	
T4	There are 4 subcategories of T4:	
	T4a	Tumour has spread into the chest wall
	T4b	Tumour has spread into the skin
	T4c	Tumour has spread into both the chest wall and the skin
	T4d	Inflammatory breast cancer
**Node**		
NX	Lymph nodes can’t be assessed	
N0	No cancer in the lymph nodes or only clusters of cancer cells < 0.2 mm	
N1	Cancer is described as one of the following:	
	N1mi	Cancer has spread to the axillary nodes and is >0.2 mm but not >2 mm
	N1a	Cancer has spread to 1–3 axillary nodes and is >2 mm
	N1b	Cancer has spread to internal mammary nodes, is >0.2 mm and found by SLNB
	N1c	Both N1a and N1b are true
N2	Cancer has spread to 4–9 axillary nodes or cancer has enlarged the internal mammary lymph nodes	
	N2a	Cancer has spread to 4–9 axillary nodes and is >2 mm
	N2b	Cancer has spread to mammary nodes and the cancer was found by imaging tests
N3		
	N3a	Cancer has spread to ≥ 10 axillary nodes and the cancer in at least one is >2 mm or the cancer has spread to the infraclavicular nodes
	N3b	Cancer has spread to 1–9 axillary nodes, the cancer in at least one of the lymph nodes is >2 mm and the cancer is found on imaging tests. Alternatively, the cancer has spread to 4–9 axillary nodes, cancer in at least one of the nodes is >2 mm, cancer has also spread to the internal mammary nodes and the cancer is >0.2 mm and found by SLNB
	N3c	Cancer has spread to the supraclavicular nodes with at least one area of cancer spread > 2 mm
**Metastasis**		
M0	No sign the cancer has spread to other parts of the body	
M1	Cancer has spread to other parts of the body, most often the bones, lungs, liver or brain	

**Table 2:** Predictive gene tests used in breast cancer care.

Name of Test	Provider	Number of Genes/Biomarkers in Test	Type of Test	Sample Required	Patient Group	Accurate with Positive Nodes?	Score	Risk Groups	Predictions	Prognostic Information
**Oncotype DX**	Exact Sciences	21	RT-qPCR	RNA from FFPE tumour tissue	Pre and post menopausal women with early HR+/HER2- breast cancer	Yes (1–3 positive nodes)	Recurrence Score (RS) 0–100	RS score < 18 is low risk, RS 18–30 intermediate risk & RS > 31 high risk	Benefit from adjuvant chemotherapy	Risk of distant recurrence after 10 years
**MammaPrint**	Agendia	70	Microarray	RNA from FFPE tumour tissue	Pre and post menopausal women with early breast cancer smaller than 5cm	Yes (1–3 positive nodes)	MammaPrint score −1 to +1	Genomic low risk (score of more than 0) or genomic high risk (score of 0 or less)	Benefit from adjuvant chemotherapy	Risk of distant recurrence after 10 years
**PAM50 (Prosigna)**	Veracyte	50	mRNA counting on nCounter Digital Analyser (NanoString)	RNA from FFPE tumour tissue	Postmenopausal women with early HR+/HER2- breast cancer who have been on ET for 5 years	Yes (1–3 positive nodes)	Risk of recurrence (ROR) score 0–100	Node-negative: low risk 0 to 40, intermediate risk 41 to 60 or high risk 61 to 100 Node-positive (up to 3): low risk 0 to 15, intermediate risk 16 to 40, or high risk 41 to 100	Benefit from adjuvant chemotherapy	Risk of distant recurrence after 10 years
**EndoPredict (EP)/EPclin**	Myriad Genetics	12	RT-qPCR	RNA from FFPE tumour tissue	Postmenopausal women with early HR+/HER2 breast cancer who have been on ET for 5 years	Yes (1–3 positive nodes)	EP score	EP score of 0 to <5 low risk, EP 5 to 15 high risk; EPclin < 3.3 low risk, EPclin ≥ 3.3 high risk	Benefit from adjuvant chemotherapy	Risk of distant and late recurrence after 10 and 15 years
**Breast Cancer Index (BCI)**	Biotheranostics	11	RT-qPCR	FFPE tumour tissue	Pre and post menopausal women with early HR+/HER2- breast cancer who have been on ET for 5 years	Yes (1–3 positive nodes)	BCI Score (0–10)	Low risk: BCI < 5; intermediate risk: BCI 5–6.4; high risk: BCI > 6.4	Benefit from extended ET	Risk of distant recurrence after 10 years
**IHC4/IHC4+C**	Not yet available	4	IHC staining + alorithm	FFPE tumour tissue	Postmenopausal women with early HR+/HER2- breast cancer who have been on ET for 5 years	Yes (1–3 positive nodes)	IHC4+C score	IHC4+C: low risk < 10%; intermediate risk 10–20%; high risk > 20%	Benefit from adjuvant chemotherapy	Risk of distant recurrence after 10 years

## Data Availability

Not applicable.
